# Supplementary material for: Multi‐dataset identification of innovative feature genes and molecular mechanisms in keratoconus
Source: J Cell Mol Med. 2024 Sep 19;28(18):e70079. doi: 10.1111/jcmm.70079 (PMC11412914; doi:10.1111/jcmm.70079)
Supplement: Supplementary file 2 — Material S2. [file JCMM-28-e70079-s002.zip › 7.GSEA analysis/2.ATOH7/KEGG_HEDGEHOG_SIGNALING_PATHWAY.html]

Details for gene set KEGG\_HEDGEHOG\_SIGNALING\_PATHWAY[GSEA]

|  || Dataset | ATOH7.group.cls#h\_versus\_l |
| Phenotype | group.cls#h\_versus\_l |
| Upregulated in class | h |
| GeneSet | KEGG\_HEDGEHOG\_SIGNALING\_PATHWAY |
| Enrichment Score (ES) | 0.46014935 |
| Normalized Enrichment Score (NES) | 1.4549674 |
| Nominal p-value | 0.015625 |
| FDR q-value | 0.27469823 |
| FWER p-Value | 0.843 |
Table: GSEA Results Summary

  

Fig 1: Enrichment plot: KEGG\_HEDGEHOG\_SIGNALING\_PATHWAY      
 Profile of the Running ES Score & Positions of GeneSet Members on the Rank Ordered List

  

| SYMBOL | TITLE | RANK IN GENE LIST | RANK METRIC SCORE | RUNNING ES | CORE ENRICHMENT || 1 | WNT1 | na | 172 | 0.697 | 0.0629 | Yes |
| 2 | WNT9A | na | 551 | 0.534 | 0.1040 | Yes |
| 3 | WNT6 | na | 825 | 0.474 | 0.1423 | Yes |
| 4 | PRKACB | na | 829 | 0.474 | 0.1883 | Yes |
| 5 | SHH | na | 937 | 0.457 | 0.2297 | Yes |
| 6 | WNT5B | na | 1090 | 0.433 | 0.2675 | Yes |
| 7 | PRKACG | na | 1784 | 0.361 | 0.2827 | Yes |
| 8 | SMO | na | 2428 | 0.314 | 0.2947 | Yes |
| 9 | WNT9B | na | 2657 | 0.300 | 0.3174 | Yes |
| 10 | BMP6 | na | 2713 | 0.298 | 0.3448 | Yes |
| 11 | BMP4 | na | 2853 | 0.288 | 0.3689 | Yes |
| 12 | WNT11 | na | 3016 | 0.280 | 0.3915 | Yes |
| 13 | BMP2 | na | 3243 | 0.268 | 0.4110 | Yes |
| 14 | WNT7A | na | 3390 | 0.261 | 0.4322 | Yes |
| 15 | WNT8B | na | 3424 | 0.259 | 0.4565 | Yes |
| 16 | GLI1 | na | 4085 | 0.233 | 0.4601 | Yes |
| 17 | DHH | na | 5410 | 0.188 | 0.4403 | No |
| 18 | WNT7B | na | 5905 | 0.174 | 0.4429 | No |
| 19 | GLI2 | na | 5995 | 0.171 | 0.4570 | No |
| 20 | WNT10A | na | 6547 | 0.156 | 0.4563 | No |
| 21 | WNT3A | na | 7555 | 0.129 | 0.4398 | No |
| 22 | WNT2 | na | 8209 | 0.114 | 0.4321 | No |
| 23 | GAS1 | na | 9944 | 0.082 | 0.3902 | No |
| 24 | CSNK1G3 | na | 10859 | 0.068 | 0.3704 | No |
| 25 | WNT4 | na | 10868 | 0.068 | 0.3768 | No |
| 26 | BMP7 | na | 11320 | 0.061 | 0.3698 | No |
| 27 | BMP5 | na | 11614 | 0.057 | 0.3669 | No |
| 28 | PRKACA | na | 12347 | 0.048 | 0.3505 | No |
| 29 | CSNK1A1L | na | 12390 | 0.048 | 0.3539 | No |
| 30 | PRKX | na | 12697 | 0.044 | 0.3493 | No |
| 31 | GLI3 | na | 14449 | 0.026 | 0.3014 | No |
| 32 | CSNK1D | na | 14704 | 0.024 | 0.2964 | No |
| 33 | WNT8A | na | 15047 | 0.021 | 0.2885 | No |
| 34 | STK36 | na | 15369 | 0.018 | 0.2810 | No |
| 35 | GSK3B | na | 16028 | 0.013 | 0.2633 | No |
| 36 | RAB23 | na | 17173 | 0.003 | 0.2306 | No |
| 37 | BTRC | na | 17413 | 0.001 | 0.2237 | No |
| 38 | SUFU | na | 17833 | -0.003 | 0.2119 | No |
| 39 | HHIP | na | 18105 | -0.005 | 0.2046 | No |
| 40 | CSNK1G2 | na | 19325 | -0.016 | 0.1709 | No |
| 41 | FBXW11 | na | 19927 | -0.021 | 0.1557 | No |
| 42 | WNT3 | na | 20041 | -0.022 | 0.1546 | No |
| 43 | PTCH2 | na | 24205 | -0.072 | 0.0415 | No |
| 44 | WNT5A | na | 24650 | -0.080 | 0.0365 | No |
| 45 | CSNK1A1 | na | 26580 | -0.117 | -0.0078 | No |
| 46 | PTCH1 | na | 26724 | -0.119 | -0.0003 | No |
| 47 | WNT16 | na | 27162 | -0.130 | -0.0003 | No |
| 48 | WNT10B | na | 27303 | -0.133 | 0.0087 | No |
| 49 | CSNK1G1 | na | 30337 | -0.223 | -0.0571 | No |
| 50 | BMP8B | na | 30368 | -0.224 | -0.0361 | No |
| 51 | WNT2B | na | 30926 | -0.244 | -0.0284 | No |
| 52 | ZIC2 | na | 31352 | -0.261 | -0.0152 | No |
| 53 | LRP2 | na | 32829 | -0.353 | -0.0234 | No |
| 54 | BMP8A | na | 33027 | -0.369 | 0.0069 | No |
| 55 | IHH | na | 33647 | -0.433 | 0.0312 | No |
Table: GSEA details [plain text format]

  

Fig 2: KEGG\_HEDGEHOG\_SIGNALING\_PATHWAY      
 Blue-Pink O' Gram in the Space of the Analyzed GeneSet

  

Fig 3: KEGG\_HEDGEHOG\_SIGNALING\_PATHWAY: Random ES distribution      
 Gene set null distribution of ES for **KEGG\_HEDGEHOG\_SIGNALING\_PATHWAY**
